# Cognitive map to support the diagnosis of solitary bone tumors in pediatric patients

**DOI:** 10.1590/0100-3984.2017.0121

**Published:** 2018

**Authors:** Felipe Costa Moreira, André Yui Aihara, Henrique Manoel Lederman, Ivan Torres Pisa, Josceli Maria Tenório

**Affiliations:** 1 Department of Health Informatics, Escola Paulista de Medicina da Universidade Federal de São Paulo (EPM-Unifesp), São Paulo, SP, Brazil.; 2 Department of Diagnostic Imaging, Escola Paulista de Medicina da Universidade Federal de São Paulo (EPM-Unifesp), São Paulo, SP, Brazil.

**Keywords:** Decision support techniques, Bone neoplasms, Child health, Diagnosis, differential, Diagnostic errors, Diagnostic imaging, Técnicas de apoio para a decisão, Neoplasias ósseas, Saúde da criança, Diagnóstico diferencial, Erros de diagnóstico, Diagnóstico por imagem

## Abstract

Abstract

**Objective:**

To present a cognitive map to support the radiological diagnosis of solitary bone tumors, as well as to facilitate the determination of the nature of the tumor (benign or malignant), in pediatric patients.

**Materials and Methods:**

We selected 28 primary lesions in pediatric patients, and we identified the findings typically associated with each of the diagnoses. The method used for the construction of the final cognitive map was the Bayesian belief network model with backward chaining.

**Results:**

We developed a logical, sequential structure, in the form of a cognitive map, based on the Bayesian belief network model, with the intention of simulating the sequence of human thinking, in order to minimize the number of unnecessary interventions and iatrogenic complications arising from the incorrect evaluation of bone lesions.

**Conclusion:**

With this map, it will be possible to develop an application that will provide support to physicians and residents, as well as contributing to training in this area and consequently to a reduction in diagnostic errors in patients with bone lesions.

## Abstract (pt)

**Objetivo:**

Apresentar um mapa cognitivo para suporte na determinação da natureza benigna ou maligna, bem como no diagnóstico radiológico, de tumores ósseos solitários na faixa etária pediátrica.

**Materiais e Métodos:**

Foi realizada uma seleção de 28 lesões principais na faixa etária pediátrica e dos achados característicos e associados a cada um desses diagnósticos. O método utilizado para a construção da estrutura cognitiva final foi o modelo de rede de crenças bayesianas com a técnica de encadeamento regressivo.

**Resultados:**

Foi desenvolvida uma estrutura lógica e sequencial no formato de um mapa cognitivo, segundo o modelo de rede de crenças bayesianas, na tentativa de simular o raciocínio humano sequencial e minimizar esforços e iatrogenias oriundas da avaliação equivocada de lesões ósseas.

**Conclusão:**

Com base nesse mapa, será possível oferecer um aplicativo para apoio a profissionais e residentes médicos e colaborar com o treinamento nessa área e, consequentemente, diminuir erros no diagnóstico de uma lesão óssea.

## INTRODUCTION

Primary malignant bone tumors constitute a minority among bone neoplasms. Although benign bone tumors are more common, their true prevalence is not known because they are frequently asymptomatic and go undiscovered—in fact, their clinical presentation can be challenging. Some lesions appear as incidental findings on routine X-rays. Depending on their appearance and the diagnostic hypothesis, the next step is to perform computed tomography or magnetic resonance imaging, as recommended by the American College of Radiology^(1)^. Normally, when dealing with a bone lesion, clinical and imaging parameters are used in order to determine the final diagnosis and, more importantly, whether the lesion is benign or malignant, with a good margin of safety.

Studies have shown that radiologists, in their evaluation of medical images, tend to use visual scanning^(2-5)^. Although visual scanning practices are comparable among radiologists with similar levels of experience, there is still broad variability in terms of the rate of diagnostic errors^(6)^. In addition, not every professional has the solid knowledge and adequate training required to combine radiological and clinical findings to confidently and logically reason out whether a lesion is benign or malignant, much less to posit a final diagnosis^(7)^. That can lead to diagnostic errors and have negative impacts, especially on the physical and psychological health of patients and their families.

Systems that support clinical and diagnostic decisions have contributed significantly to the management of medical knowledge, facilitating processes and the use of knowledge, from investigation and diagnosis to treatment and long-term care. Their role and acceptance in daily clinical practice is on the rise. The computerization of clinical guidelines has been drawing increased interest in recent years due to its facilitating their dissemination and improving the processes based on the knowledge by which they were created^(8)^.

Cognitive maps have contributed to part of the evolution of decision support systems. According to Bougon^(9)^, cognitive map is the generic term used in order to represent possible patterns of relationships between concepts. The words and phrases used by individuals to express ideas and concepts in a given context are the building blocks of a cognitive map. Swan^(10)^ makes a distinction between the maps and the mapping techniques. According to that author, cognitive mapping is understood as a set of techniques or research tools to identify the elements that make up these maps or models built by individuals and shared, to a greater or lesser degree, with others. A cognitive map is used in order to identify the values of an individual or group and to reduce the antagonism between these values. Its ability to capture multiple values and reduce their conflicting aspects provides the logic to analyze the decision-making problems of interested parties^(11)^.

The objective of this study was to present a cognitive map to support radiological diagnoses and to determine the benign or malignant nature of solitary bone tumors in children and adolescents up to 19 years of age^(12)^. We constructed this map by means of a Bayesian belief network model^(13)^, using multi-criteria sequential decision making based on specific clinical and radiological attributes that can be obtained by conventional radiography, computed tomography, and magnetic resonance imaging.

## MATERIALS AND METHODS

We constructed the final cognitive map by using the Bayesian belief network model^(13)^ with the backward chaining technique^(14)^. A Bayesian network—a network of beliefs or a directed acyclic graphical model—is a probabilistic graphical model that represents a set of variables (nodes) and their conditional interdependencies. The nodes can represent observable medical data such as imaging findings, clinical attributes, etc., whereas the interconnected edges can support measures of quantitative or qualitative origin to describe a given relationship. Nodes can either be known with certainty or described as uncertainties using a subjective probability. Subjective probabilities express the degree of belief, taking into account the baseline knowledge of the individual. This notion of probability differs from the more commonly used classical probability^(15,16)^.

The construction of a belief network follows a common set of guidelines^(13)^, such as including all concepts (input and decision concepts) for the modeling of systems, using causal knowledge to assign interconnections in the graph, and using prior knowledge to specify conditional distributions (probabilities). Backward chaining starts with the final attribute (the diagnosis) and seeks only the values of the variables necessary for its deduction, processing only what is relevant to obtain a diagnosis^(14)^.

**Figure 1:**
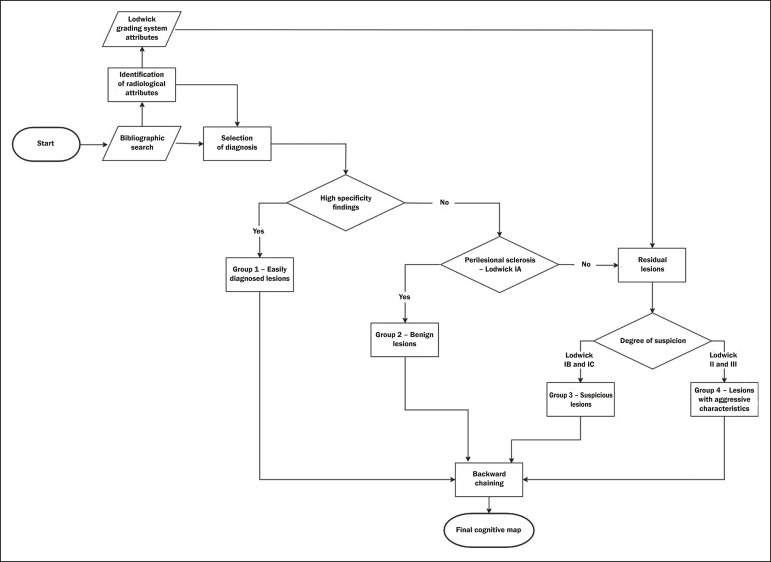
Sequential representation of the steps for the construction of a cognitive map.

The steps for constructing a cognitive map consist of acquiring knowledge, selecting diagnoses, grouping the lesions, and backward chaining (Figure 1). To begin building this structure, knowledge was acquired by searching the literature to find the main bone tumors that were part of the list of distinctions within the pediatric population^(1,17-22)^. As shown in Table 1, we then selected the lesion types that were most common in the ≤ 19-year age group—23 types of benign lesions and 5 types of malignant lesions—identifying their key distinctions, even if the incidence in pediatric patients was low^(21)^.

**Table 1:** List of selected diagnoses in the pediatric population, by nature.

Nature	Diagnoses
Benign	Aneurysmal bone cyst, simple bone cyst, subchondral cyst, fibrous cortical defect, periosteal desmoid, fibrous dysplasia, enchondroma, enostosis, chondromyxoid fibroma, non-ossifying fibroma, stress fracture, insufficiency fracture, intraosseous ganglion, synovial herniation, Langerhans cell histiocytoma, bone infarction, Freiberg's infraction, osteoblastoma, osteochondroma, osteoma, osteoid osteoma, osteomyelitis, giant cell tumor
Malignant	Chondrosarcoma, conventional osteosarcoma, telangiectatic osteosarcoma, periosteal osteosarcoma, Ewing's sarcoma

We included the most relevant diagnoses and those that had a greater impact on the follow-up and health status of patients, such as malignant bone tumors, which, although rare, are associated with high rates of morbidity and mortality. Lastly, we included lesions with highly specific (pathognomonic) findings, which, when present, even with a low incidence among pediatric patients, could determine the diagnosis with certainty, such as stress fractures and Freiberg’s infraction (Table 2). Characteristically nonspecific lesions were not included, because of their different forms of presentation in imaging studies, in which they can mimic other tumors.

**Table 2:** Diagnoses and their specific findings.

Diagnosis	Specific finding
Subchondral cyst	Subchondral location with arthrosis
Intraosseous ganglion	Subchondral location without arthrosis
Fibrous dysplasia	Ground-glass opacity
Enostosis	Highly dense (similar to cortical bone) in the bone marrow
Osteoma	Highly dense (similar to cortical bone) on the cortical bone itself
Bone infarction	Peripheral serpentine line

After selecting the tumors, we identified characteristic findings associated with each radiological diagnosis (Table 3). The terms used were chosen according to the selected bibliography^(1,17-21)^. The main radiographic characteristics that should be evaluated for a bone lesion are location (longitudinal or transverse), margins and transition zone, periosteal reaction, mineralization, size, number of lesions (this was not applied in the present study, because it deals only with the assessment of solitary lesions), and the presence of a soft tissue component^(22)^. We also considered radiological findings such as contrast enhancement and location in the skeleton, as well as findings with a high degree of specificity for certain lesions, such as formation of fluid-fluid level, ground-glass opacity, serpentine line, dense sclerotic lesions, and subchondral location. Some clinical findings were also considered in order to support and increase the specificity of the final diagnosis.

**Table 3:** Main radiological attributes associated with bone tumors.

Category	Attributes
Main radiological attributes	Density/intensity, presence of osteoarthrosis, longitudinal location, transverse location, perilesional sclerosis, open or closed physis, chondral matrix, size (greater or smaller than 1.5 cm), definition of margins, geographic/mottled aspect
	Periosteal reaction, contrast enhancement, edema/ adjacent medullary sclerosis, location in the skeleton
Other radiological attributes	Peripheral serpentine line, ground-glass opacity, formation of a fluid-fluid level, septations, exostosis, transverse linear aspect
Clinical support attributes	Asymptomatic, acute or chronic pain, signs of inflammation, fever, anemia, palpable mass, relationship with physical activity

Initially, we separated the lesions related to the high-specificity findings that are associated with only one (pathognomonic) diagnosis or two possible diagnoses. A conceptual assessment was then made of the Lodwick grading system for tumor aggressiveness^(23)^, as detailed in Table 4. This system is widely used in the prediction of the growth rate for lytic bone lesions and considers the following criteria to be the most relevant aspects for radiological staging: bone destruction, definition of margins, marginal sclerosis, cortical expansion (larger or smaller than 1 cm), and cortical penetration. Of those criteria, marginal sclerosis is the only one that can be found in grade IA (low grade; not aggressive); it was therefore considered the most important criterion (higher degree of specificity) to identify benign lesions, also known as “do not touch” lesions. The others were combined to form two other groupings, one with possibly benign lesions corresponding to Lodwick grades IB and IC (medium grade; moderately aggressive), and another group with definitely suspicious lesions, corresponding to Lodwick grades II and III (high grade; very aggressive).

**Table 4:** Lodwick grading system^(23)^.

Radiological characteristics	Aggressiveness	Classification
Geographic destruction; well-defined radiolucency; with perilesional sclerosis; cortical expansion up to 1 cm	Low grade/non-aggressive	I-A
Geographic destruction; well-defined radiolucency; with no perilesional sclerosis; cortical expansion greater than 1 cm	Medium grade/moderately aggressive	I-B
Geographic destruction; complete cortical penetration; poorly-defined margins	Medium grade/moderately aggressive	I-C
Geographic destruction with mottled/infiltrative aspect; irregular margins of medium size; poorly-defined outlines	High grade/very aggressive	II
Geographic destruction with isolated mottled/ infiltrative aspect; numerous erosions parallel to the long axis of the bone	High grade/very aggressive	III

After dividing the lesions into four groups, the other radiological findings were applied to each group in succession, starting with the lower specificity criteria and progressing to those with higher specificity. Thus, we created a backward chaining model in which criteria are ordered by their specificity up until the final diagnosis (higher specificity).

We used CMapTools, a free and open-source software for the graphical construction of a linear, acyclic structure using nodes and edges, structured as a decision tree, with clinical and radiological attributes. After the construction of the cognitive map, two experienced radiologists—one specializing in musculoskeletal radiology and the other specializing in pediatric radiology, with 21 and 44 years of professional activity, respectively—were consulted for validation and adjustments to the final cognitive map, with radiological and clinical concepts applied to the diagnosis of pediatric bone tumors.

## RESULTS

**Figure 2:**
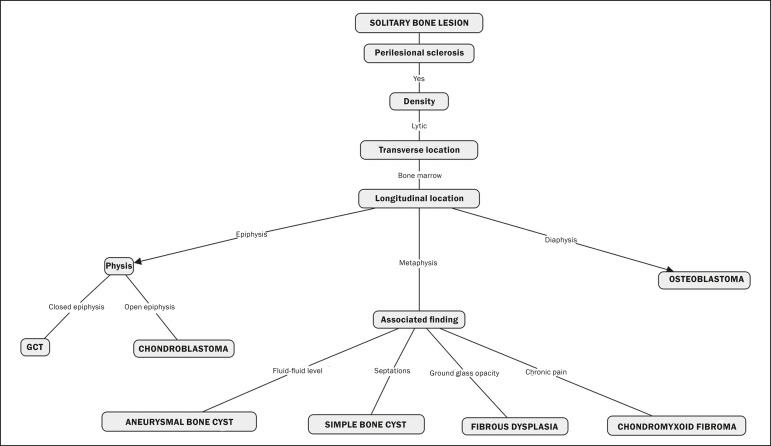
Branch of the cognitive map constructed, partially representing the group of benign lesions. GCT, giant cell tumor.

The map starts with an indeterminate solitary bone lesion. Branching first occurs with lesions that are easily identifiable and that have characteristic findings. At this point, it subdivides into three branches: one for well-defined blastic lesions such as osteoma, enostosis, fibrous dysplasia, and metastases from the breast or prostate; another for a small group of epiphyseal lytic lesions typically located subchondrally, including degenerative subchondral cysts and intraosseous ganglion cysts; and the third containing only bone infarction with its characteristic finding of a peripheral serpentine line. If none of these characteristics is present, the map progresses to the next node, which divides into two major branches: one for lesions with perilesional sclerosis and another for those without. The process of backward chaining begins with the branch for lesions with perilesional sclerosis and leads to the final diagnoses, as can be observed in one of its main branches (Figure 2). On the basis of the remaining Lodwick grading system criteria, the branch containing lesions without perilesional sclerosis is in turn subdivided into two other branches—one for suspicious lesions and the other for lesions with aggressive characteristics. The group of suspicious lesions begins the backward chaining process. However, before the backward chaining process, the branch of lesions with aggressive characteristics passes through a filter to separate some benign lesions that manifested radiologically with attributes of aggressiveness but which, when associated with certain findings or at certain locations in the skeleton, can be diagnosed or suggested without much difficulty, such as stress/insufficiency fractures and Freiberg’s infraction.

## DISCUSSION

The decision and the reasoning behind the proper diagnosis of bone cancer are complex and involve subjective decision criteria and a variety of possibilities. In addition to this complexity, diagnostic errors can occur for various reasons that involve the professional, the work environment, or both^(24)^. These diagnostic errors can be harmful to patients and their families. They can also have economic consequences for those involved, such as public agents, health plans, health professionals, etc.^(25)^, because they require new diagnostic approaches that are potentially unnecessary, not to mention the legal consequences of such errors. In this context, it is worth noting that more important than the final diagnosis is the definition of the nature or degree of aggressiveness of a bone lesion. In fact, it is on the basis of this definition that decisions will be made regarding the follow-up, the need for new imaging techniques, or any recommendations for surgery or a biopsy.

The present study is not the first to present a framework for the classification of tumor lesions. However, the number of studies that have correlated the application of Bayesian networks to cancer is limited. In addition, most published results come from preliminary studies that are based on patient data^(26)^.

Forsberg et al.^(27)^ conducted a study to determine the feasibility of developing Bayesian classifiers for estimating survival in patients undergoing surgery for axial and appendicular skeletal metastases. To do this, they developed and trained a Bayesian network model to estimate survival in months, using characteristics based on patients’ data. Kharya et al.^(28)^ conducted a study on the use of Bayesian networks for breast cancer. That type of model is appropriate because of its ability to create a symbolic representation and to manipulate the uncertainty regarding the likelihood of various scenarios according to the evidence given. The authors investigated the usefulness of such a network for automated detection of breast cancer and found it to be a potentially useful technique.

In our study, we developed a cognitive map following a well-defined mapping method using a Bayesian network model that could emulate the logical and sequential reasoning needed to diagnose pediatric bone tumors. We believe that this map can help minimize efforts and errors in defining the aggressiveness of a lesion and in suggesting a final diagnosis. It can also help less-experienced radiologists by providing an organized and systematic structure for logical critical reasoning. For example, such a map can support the formulation of more cogent hypotheses and guide orthopedic physicians in the management and treatment of unknown lesions.

One major limitation of this study is that not all possible pediatric bone tumors could be included, which reduces its power and accuracy. There are also other limitations, such as the acyclic characteristic of the model itself, impeding lateral connections between the various branches of the tree—as would normally occur with graphic networks—and preventing diagnoses from different branches from being presented together when there is a combination of findings other than those predicted and pre-formulated on the map. In other words, the cognitive map proposed here is a prototype of a deterministic and limited combination of tumors and their respective attributes.

In the literature consulted, we found no clear definitions of the degrees of specificity and sensitivity of the radiological findings of bone tumors, which, in and of itself, prevented the use of a quantitative approach to the distribution and organization of the multiple criteria. Therefore we adopted a predominantly qualitative, subjective method for constructing a cognitive map. For future studies, we believe that a statistical survey presenting the exact degree of specificity of each finding in relation to certain tumors would be useful, because such studies would then be able to include probabilities in the various levels and branches of the map.

An application is being developed that can assist a user in navigating forward or backward along the branches of the tree. A teaching mechanism consisting in presenting information and theoretical content along with the nodes and edges of the map, will also be introduced. There are evaluations planned with groups of medical residents in order to measure the accuracy of the map and its acceptance as a reasoning support to facilitate the diagnosis of pediatric bone lesions.

## CONCLUSION

The complexity of the decision-making and reasoning processes in diagnosing bone cancer can generate countless adverse effects that arise from iatrogenic complications. Not all professionals have access to the training and education that would give them the proper logical and clinical insight to correctly deal with a bone lesion. The present study proposes a cognitive map to support the radiological diagnosis of solitary bone tumors in pediatric patients. With this map, it will be possible to develop an application that will provide support to physicians and residents, as well as contributing to training in this area, thereby leading to a reduction in diagnostic errors in the evaluation of bone lesions.
